# Comparative evaluation of the quality, reliability, and readability of five large language model responses to frequently asked questions on gestational hypertension

**DOI:** 10.3389/fpubh.2026.1833611

**Published:** 2026-05-29

**Authors:** Zhongyu Liu, Pengfei Tong, Yujie Jiang, Rong Liu

**Affiliations:** 1 Department of Pharmacy, The Affiliated Changsha Central Hospital, Hengyang Medical School, University of South China, Changsha, China; 2 International Advanced Technology Application Promotion Center, Hefei, China; 3 Emergency Department, Affiliated Jinhua Hospital, Zhejiang University School of Medicine, JinHua, China

**Keywords:** artificial intelligence, gestational hypertension, large language models, online medical information, quality, readability

## Abstract

**Objective:**

This study aims to clarify the impact of Large Language Models (LLMs) and health education content categories on generated text quality (patient education appropriateness and overall quality) and readability, providing empirical evidence for the standardized application of LLMs-assisted health communication.

**Methods:**

Five mainstream models (Doubao, Deep Seek, Wenxin Yiyan, Gemini and GPT-5) were selected to generate 100 texts (20 per model, 20 per theme) across five health education categories: disease cognition dimension, etiology and risk factors dimension, diagnosis and examination dimension, treatment and management dimension, and prevention and prognosis dimension. Test quality was assessed using the Chinese version of the Patient Education Material Readability Assessment Scale (C-PEMAT) and the Global Quality Scale (GQS), while readability was measured via seven metrics including the Automated Readability Index (ARI) and the Flesch Reading Ease Score (FRES). Correlation analyses were used to explore relationships among indicators.

**Results:**

Our analysis revealed clear hierarchical performance across five large language models: GPT-5 achieved the highest scores in both patient education appropriateness (C-PEMAT: 11.10 ± 2.40) and overall text quality (GQS: 5.00 [4.00, 5.00]). GPT-5 exhibited significantly higher GQS scores than all other models (*χ*^2^ = 66.52, *p* < 0.001), while Wenxin Yiyan ranked lowest in core quality (GQS: 1.00 [1.00, 2.00]). Content categories exhibited differentiated readability but stable quality: texts on “Prevention and Prognosis” and “Treatment and Management” yielded the highest C-PEMAT scores, whereas “Etiology and Risk Factors” texts showed weaker reading fluency. Correlation analysis confirmed that quality and readability were largely independent, though subtle associations emerged—including a weak positive link between FRES and GQS. In the factual-accuracy assessment, 19.0% of responses contained factual inaccuracies, while no response was judged to contain potentially clinically harmful misinformation. Significant between-model differences were observed in factual accuracy scores.

**Conclusion:**

This study demonstrates significant hierarchical performance among LLMs in health science text creation. Different health education themes show partial indicator variation but stable overall quality. Notably, quality and readability are relatively independent (with weak correlations), providing empirical evidence for understanding LLMs in health popularization.

## 1 Introduction

Hypertensive disorders in pregnancy (HDP) affect approximately 10% of pregnancies globally and are a leading cause of maternal, fetal, and neonatal morbidity and mortality (1, 2). As maternal age and obesity rise, these conditions are expected to become even more prevalent (3). Clinically, HDP are categorized into four subtypes based on the onset of hypertension and presence of target organ involvement: chronic hypertension, preeclampsia, gestational hypertension, and chronic hypertension with superimposed preeclampsia (1, 4). Pregnant women with HDP face an elevated risk of placental abruption, stroke, pulmonary edema, thromboembolic events, disseminated intravascular coagulation, and multiple organ failure (5, 6). For the fetus, associated risks include intrauterine growth restriction, preterm birth, and intrauterine death—all of which are notably heightened in preeclampsia (7). Numerous studies have identified second-trimester angiogenic factors (soluble fms-like tyrosine kinase 1 [sFlt-1], placental growth factor [PlGF], and soluble endoglin) as promising tools for predicting early-onset preeclampsia (8, 9). However, no single test can reliably predict preeclampsia to date, and further prospective investigations are warranted to validate its clinical utility. Emerging evidence indicates that pregnant women’s cognitive understanding of HDP and associated psychological distress significantly influence the incidence of adverse pregnancy outcomes and treatment adherence (10). Thus, delivering scientific, accurate, and accessible health education is of paramount importance. Early diagnosis and optimal management of hypertensive pregnant women remain clinical priorities, as these measures markedly improve maternal and fetal outcomes. In the digital era, the internet has become the primary channel for pregnant women to access health information. However, the quality of online medical content varies greatly, and many materials are too technical, poorly structured, or inconsistent with clinical guidelines. Such low-quality information may cause misunderstanding, anxiety, or even inappropriate self-management among patients.

Artificial intelligence (AI) is formally defined as the study of algorithms that endow machines with reasoning capabilities and cognitive functions, including object and word recognition, problem-solving, and decision-making (11–13). In recent years, AI-powered chatbots and virtual assistants have been increasingly adopted in healthcare to interact with patients, answer queries, and deliver basic medical information (11, 14, 15). A typical example is ChatGPT-3.5, which has gained worldwide popularity for its natural language generation ability (16). Meanwhile, a variety of large language models (LLMs) have also been developed and applied rapidly in medical scenarios (11, 17, 18). Despite the potential of AI to improve healthcare quality and safety, concerns remain about the reliability, quality, and readability of its outputs (12, 13, 19, 20). Low-quality or inaccurate AI-generated content risks misleading patients and causing potential health risks. In addition, many online materials are too complex for the general public to understand. Leading health authorities—including the National Institutes of Health (NIH), the American Medical Association (AMA), and the U.S. Department of Health and Human Services (HHS)—recommend that patient-facing materials be written at or below a sixth-grade reading level to ensure accessibility (21–23). Against this backdrop, the systematic assessment of readability and quality for AI-generated HDP patient education materials is of paramount clinical and public health importance.

To address these critical gaps, this study evaluates the reliability, quality, and readability of responses from five leading LLMs—Doubao, DeepSeek, Wenxin Yiyan, Gemini, and GPT-5—to frequently asked questions about HDP. Our findings delineate the respective strengths and limitations of these LLMs in addressing patient-centric HDP inquiries, while identifying critical areas for optimization and practical implementation challenges. This research further underscores the pivotal role of LLMs in enhancing patient engagement and health literacy to support clinical management of HDP, ultimately offering evidence-based support for the targeted use of AI in maternal health education.

## 2 Materials and methods

### 2.1 Ethical considerations

This study was executed in silico without human or animal involvement or any patient-related information; accordingly, Institutional Review Board approval was not required.

### 2.2 Research procedure

On November 25, 2025, two clinical experts compiled a total of 20 common questions about gestational hypertension. These 20 questions were divided into five aspects (Disease Cognition, Etiology and Risk Factors, Diagnosis and Examination, Treatment and Management, and Prevention and Prognosis). For detailed content, please refer to Table 1. Researchers input the 20 questions into publicly accessible large language models: Doubao,^1^ DeepSeek,^2^ Wenxin Yiyan,^3^ Gemini^4^ and GPT-5.^5^ The five large language models were selected based on four core criteria: (1) public accessibility and mainstream popularity; (2) regional representativeness (three Chinese domestic models and two international models); (3) frequent application in online health information services; and (4) typical performance stratification in general-purpose question answering. This selection ensures the study reflects real-world usage and comparative validity. The answered questions are analyzed from three dimensions: readability, reliability and quality. Identical, standardized neutral prompts were used for all models to ensure output comparability. Prompts were kept consistent in structure, wording, and length without customization per model.

**Table 1:** Issue list.

Issue list
Disease cognition
1. What is the definition of gestational hypertension and during what period of pregnancy is it diagnosed? 2. What are the main differences between gestational hypertension, preeclampsia, and chronic hypertension? 3. What are the common clinical manifestations and signs of gestational hypertension? 4. What are the main potential harms of gestational hypertension for the mother and the fetus?
Etiology and risk factors
1. What are the main pathophysiological mechanisms underlying gestational hypertension? 2. Which groups are at high risk for gestational hypertension, and what are the key risk factors? 3. How does pregnancy affect the risk for women with a history of hypertension or kidney disease? 4. Do obesity, diabetes, and advanced maternal age increase the risk of gestational hypertension?
Diagnosis and examination
1. What are the blood pressure diagnostic criteria and measurement requirements for gestational hypertension? 2. How can gestational hypertension, preeclampsia, and chronic hypertension during pregnancy be distinguished? 3. Which laboratory tests and auxiliary examinations are needed for patients with gestational hypertension? 4. What are the main indicators used to assess disease severity and progression to severe preeclampsia?
Treatment and management
1. What are the first-line medications and basic principles for blood pressure control in gestational hypertension? 2. How do the management strategies differ between mild gestational hypertension and severe preeclampsia? 3. Under what circumstances is hospitalization or even early termination of pregnancy required? 4. What issues should be noted regarding medication safety and monitoring in patients with gestational hypertension?
Prevention and prognosis
1. What are the key preventive measures to reduce the risk of gestational hypertension? 2. What is the preventive role of low-dose aspirin supplementation in high-risk pregnant women? 3. How should diet and lifestyle be adjusted in patients with gestational hypertension?4. How should postpartum follow-up and long-term cardiovascular risk monitoring be carried out in women with a history of gestational hypertension?

The initial 20 questions were developed in Chinese. These Chinese questions were professionally translated into English by an obstetrics and gynecology expert and a clinical expert. Any discrepancies were resolved through consensus prior to analysis. The English prompts were subsequently input into five large language models to generate English responses. All readability analyses were conducted directly on the original English responses. For quality assessment using the Chinese version of the Patient Education Material Readability Assessment Tool (C-PEMAT), all English responses were translated back into Chinese by the same two experts.

### 2.3 Readability evaluation

We employed multiple formulas from the Text Readability Assessment Tool^6^ to analyze responses generated by LLMs. The formulas used in text readability were Linsear Write (LW), Coleman-Liau Readability Index (CLI), Automated Readability Index (ARI), Simple Measure of Gobbledygook (SMOG), Gunning Fog Readability (GFOG), The Flesch Reading Ease Score (FRES) and Flesch–Kincaid Grade Level (FKGL). Final readability scores were recorded as median (minimum-maximum). These indicators are used to evaluate the readability and understandability of the text generated by LLMs to everyday spoken language.

### 2.4 Quality assessment

In this study, the Chinese version of the Patient Education Material Readability Assessment Scale (C-PEMAT) and the Global Quality Score (GQS) were employed to evaluate the quality of LLM-generated responses. The C-PEMAT comprises 24 indicators categorized into two dimensions: “Comprehensibility” (16 items, including logical information organization and accessibility of specialized terminology) and “Practicality” (8 items, including specific action guidance and suitability for target audiences). Each indicator is scored on a 0–1 scale (0 = completely non-compliant, 1 = completely compliant), yielding a total score ranging from 0 to 24. Higher scores indicate better accessibility for end users. The GQS is a 1–5 ordinal rating scale used to assess the overall quality of online health information: 1 = poor quality, not useful to patients; 2 = low quality, very limited utility; 3 = fair quality, somewhat useful; 4 = good quality, useful; 5 = excellent quality, highly useful to patients.

On November 25, 2025, two independent clinical experts with more than 2 years of relevant clinical experience scored all 100 texts. Two independent raters evaluated all 100 model-generated responses. For C-PEMAT, each response was assessed using 24 binary items. The 24 item scores were summed to obtain a total C-PEMAT score for each text. Inter-rater reliability for C-PEMAT was calculated at the text level using these total scores, rather than at the individual item level or by averaging *κ* values across the 24 items. For GQS, which uses a 1–5 ordinal scale, inter-rater reliability was assessed using quadratic weighted κ at the text level. Cohen’s κ or weighted κ values are reported with 95% confidence intervals.

Of the 100 evaluated texts, three required adjudication by a third expert due to initial scoring discrepancies between the two raters. All discrepancies were resolved via face-to-face consensus discussion to ensure the rigor of the assessment. Inter-rater reliability was interpreted using the following criteria: κ > 0.75 indicated excellent agreement; 0.40 ≤ κ ≤ 0.75 indicated acceptable agreement; and κ < 0.40 indicated poor agreement. Subsequent verification confirmed that both C-PEMAT and GQS achieved excellent inter-rater reliability, with κ values greater than 0.75.

### 2.5 Factual-accuracy assessment

To further support the assessment of reliability, all 100 LLM-generated responses were evaluated for factual accuracy by an obstetrician-gynecologist with reference to current clinical guidance on hypertensive disorders of pregnancy. A 5-point Likert accuracy scale was used: 5 indicated completely accurate information with no factual errors; 4 indicated generally accurate information with only minor omissions or imprecision; 3 indicated partially accurate information with factual inaccuracies but no direct clinical harm; 2 indicated potentially misleading information that could lead to inappropriate understanding or health behavior; and 1 indicated clearly incorrect or clinically harmful information. Responses with an accuracy score of 3 or lower were considered to contain factual inaccuracies, whereas responses with an accuracy score of 2 or lower were considered to contain potentially clinically harmful errors.

### 2.6 Statistical analysis

In this study, statistical methods were selected based on the distribution characteristics of the data. For normally distributed parameters (such as C-PEMAT scores), we report means with standard deviations (M ± SD) and used one-way ANOVA for comparing multiple groups. For non-normally distributed metrics (e.g., GQS scores, as well as ARI and FRES scores), medians and interquartile ranges [M (Q₁, Q₃)] are presented, with group comparisons performed using the Kruskal–Wallis H test. A *p* value of < 0.05 was considered to indicate a significant difference. All data analyses and visualizations were performed using Python 3.14.

## 3 Results

### 3.1 Factual-accuracy assessment

A factual-accuracy assessment was conducted for all 100 LLM-generated responses. Overall, 23 responses were rated as completely accurate, 58 were rated as generally accurate with only minor omissions or imprecision, and 19 were rated as containing factual inaccuracies without direct clinical harm. Therefore, the proportion of responses containing factual inaccuracies, defined as an accuracy score of 3 or lower, was 19.0%. No response was rated as containing potentially clinically harmful misinformation, defined as an accuracy score of 2 or lower; therefore, the proportion of clinically harmful responses was 0.0%. Detailed results for each model are presented in Table 2.

**Table 2:** Factual accuracy scores across different models.

Model	Mean ± SD	Median, IQR	Acc = 5, n/N (%)	Acc ≤ 3, n/N (%)	Acc ≤ 2, n/N (%)
GPT-5	4.85 ± 0.37	5.00, 5.00–5.00	17/20, 85.0%	0/20, 0.0%	0/20, 0.0%
Doubao	4.15 ± 0.49	4.00, 4.00–4.00	4/20, 20.0%	1/20, 5.0%	0/20, 0.0%
DeepSeek	4.10 ± 0.31	4.00, 4.00–4.00	2/20, 10.0%	0/20, 0.0%	0/20, 0.0%
Gemini	4.00 ± 0.00	4.00, 4.00–4.00	0/20, 0.0%	0/20, 0.0%	0/20, 0.0%
Wenxin Yiyan	3.10 ± 0.31	3.00, 3.00–3.00	0/20, 0.0%	18/20, 90.0%	0/20, 0.0%
Overall	4.04 ± 0.65	4.00, 4.00–4.00	23/100, 23.0%	19/100, 19.0%	0/100, 0.0%

Kruskal–Wallis test revealed highly significant differences in factual accuracy scores across models (H = 73.17, *p* < 0.001).

The factual accuracy score differed significantly among the five models according to the Kruskal–Wallis test (H = 73.17, *p* < 0.001). In this dataset, GPT-5 showed the highest median factual-accuracy score, with a median score of 5.00, IQR 5.00–5.00, whereas Wenxin Yiyan showed the lowest median factual-accuracy score, with a median score of 3.00, IQR 3.00–3.00.

### 3.2 Readability analysis

This study focused on the impacts of large language model (LLM) types and health education content categories on the quality and readability of generated texts. We first evaluated five leading models (Doubao, DeepSeek, Wenxin Yiyan, Gemini, and GPT-5) using three criteria: patient-education appropriateness (C-PEMAT), overall text quality (GQS), and a battery of readability indices (ARI, FRES, GFOG, FKGL, CL, SMOG, LW). We then applied the same assessment framework to texts organized under five core health-education themes: disease cognition, etiology and risk factors, diagnosis and examination, treatment and management, and prevention and prognosis. Through multi-dimensional analysis, this study clarifies how model types and content categories impact the quality and readability of generated texts, thereby providing empirical support for the targeted application of AI in health science communication.

At the model level, capabilities show clear stratification: GPT-5 stands out as the top performer in core quality. Its C-PEMAT score (11.10 ± 2.40) is the highest across all models—30.6% higher than Wenxin Yiyan (8.50 ± 2.69). GPT-5 showed the highest median GQS score [5.00 (4.00, 5.00)], whereas Wenxin Yiyan showed the lowest [1.00 (1.00, 2.00)]. Doubao exhibits a “high readability, low syntactic complexity” profile: its median FRES readability score (31.50) ranks first among all models, 142% higher than Gemini’s (13.00, the lowest). By contrast, its median GFOG (14.90) and median CL (14.84) are relatively low, meaning its texts are highly fluent to read but feature simpler sentence structures. Deep Seek [C-PEMAT: 9.10 ± 2.00; GQS: 3.00 (3.00, 4.00)] and Gemini [C-PEMAT: 9.35 ± 2.13; GQS: 2.00 (1.00, 3.00)] fall into the middle tier. Notably, Gemini has notably high values in several readability indicators (median GFOG: 17.60; median FKGL: 18.14), reflecting greater linguistic complexity. Wenxin Yiyan ranks lowest in core quality, with a GQS score significantly lower than other models; while it performs moderately in some readability metrics, it offers limited practical value overall. Additionally, significant differences exist between models in GFOG (*χ*^2^ = 11.74, *p* = 0.019) and CL (*χ*^2^ = 15.08, *p* = 0.005), further confirming that models differ distinctly in syntactic complexity. These results are detailed in Table 3.

**Table 3:** Performance comparison of different large language models.

Variables	Total (*n* = 100)	Doubao (*n* = 20)	Deep Seek (*n* = 20)	Wenxin Yiyan (*n* = 20)	Gemini (*n* = 20)	GPT-5 (*n* = 20)	Statistic	*p*
C-PEMAT score, Mean ± SD	9.34 ± 2.39	8.65 ± 1.90	9.10 ± 2.00	8.50 ± 2.69	9.35 ± 2.13	11.10 ± 2.40	*F* = 4.31	0.003
GQS score, M (Q₁, Q₃)	3.00 (2.00, 4.00)	3.00 (2.75, 4.00)	3.00 (3.00, 4.00)	1.00 (1.00, 2.00)	2.00 (1.00, 3.00)	5.00 (4.00, 5.00)	χ^2^ = 66.52#	< 0.001
ARI, M (Q1, Q3)	19.09 (16.45, 22.13)	17.84 (15.46, 24.31)	18.48 (16.45, 21.79)	17.95 (16.02, 20.44)	20.19 (19.05, 21.02)	19.34 (15.92, 23.60)	χ^2^ = 4.47#	0.346
FRES, M (Q1, Q3)	17.00 (8.00, 34.00)	31.50 (19.50, 40.50)	14.00 (5.25, 26.25)	16.00 (10.75, 31.00)	13.00 (7.75, 17.00)	22.00 (12.00, 36.25)	χ^2^ = 8.04#	0.09
GFOG, M (Q1, Q3)	16.35 (14.25, 18.30)	14.90 (13.10, 16.45)	17.30 (14.45, 20.40)	17.15 (16.00, 18.93)	17.60 (15.93, 18.57)	15.90 (13.60, 16.40)	χ^2^ = 11.74#	0.019
FKGL, M (Q1, Q3)	16.26 (14.59, 19.21)	15.46 (13.27, 19.98)	15.61 (14.54, 19.12)	16.02 (14.83, 18.62)	18.14 (17.20, 19.09)	17.62 (13.42, 20.40)	χ^2^ = 3.87#	0.424
CL, M (Q1, Q3)	16.63 (14.76, 18.21)	14.84 (13.97, 16.65)	17.25 (16.43, 20.24)	16.95 (14.85, 18.55)	17.36 (16.58, 18.35)	15.60 (14.35, 16.70)	χ^2^ = 15.08#	0.005
SMOG, M (Q1, Q3)	14.62 (12.48, 16.26)	13.38 (12.40, 15.53)	13.32 (12.26, 16.76)	13.72 (12.43, 15.75)	15.41 (14.87, 16.63)	14.39 (12.37, 16.26)	χ^2^ = 4.39#	0.356
LW, M (Q1, Q3)	53.00 (49.00, 59.00)	57.00 (52.75, 60.50)	52.00 (48.50, 59.25)	54.00 (49.00, 57.00)	50.00 (46.00, 55.75)	54.50 (48.50, 58.00)	χ^2^ = 5.71#	0.222

F: ANOVA, #: Kruskal–Wallis test. SD, standard deviation; M, Median; Q1, 1st Quartile; Q3, 3rd Quartile.

The content category dimension is defined by “differentiated readability indicators, stable quality”: C-PEMAT (health information readability) shows extremely significant differences (*F* = 11.59, *p* < 0.001). The “Prevention and Prognosis” (11.20 ± 1.82) and “Treatment and Management” (10.75 ± 2.02) categories score highest—40.0 and 34.4% higher than the lowest-performing “Etiology and Risk Factors” category (8.00 ± 1.78), respectively—proving these two text types better meet the public’s need to understand health information. LW (word length) also shows significant differences (χ^2^ = 12.82, *p* = 0.012): the “Prevention and Prognosis” category has the longest median (58.00), while the “Treatment and Management” category has the shortest (49.50)—an 8.5-point gap, indicating notable variations in text length across themes. The SMOG index also reveals significant inter-group differences (χ^2^ = 9.82, *p* = 0.044). Though readability indicators like ARI and FKGL do not reach statistical significance (*p* > 0.05), they show a clear trend: higher values in the “Treatment and Management” category and lower values in the “Etiology and Risk Factors” category. Specifically, the “Treatment and Management” category has the highest median ARI (22.35) and median FKGL (19.39), meaning its texts are more linguistically complex; the “Etiology and Risk Factors” category has a lower median FRES (13.50), suggesting weaker reading fluency. In contrast, GQS (overall quality) shows no statistical differences across themes (χ^2^ = 2.54, *p* = 0.637), demonstrating that AI can maintain consistently professional text quality across different content dimensions. Corresponding data are presented in Table 4.

**Table 4:** Analysis results by content category.

Variables	Total (*n* = 100)	Disease cognition dimension (*n* = 20)	Etiology and risk factors dimension (*n* = 20)	Diagnosis and examination dimension (*n* = 20)	Treatment and management dimension (*n* = 20)	Prevention and prognosis dimension (*n* = 20)	Statistic	*p*
C-PEMAT score, Mean ± SD	9.34 ± 2.39	8.10 ± 1.74	8.00 ± 1.78	8.65 ± 2.52	10.75 ± 2.02	11.20 ± 1.82	F = 11.59	<0.001
GQS score, M (Q₁, Q₃)	3.00 (2.00, 4.00)	3.00 (2.75, 4.00)	3.50 (1.75, 4.00)	3.00 (2.00, 4.00)	2.00 (2.00, 3.25)	3.00 (1.00, 4.00)	χ^2^ = 2.54#	0.637
ARI, M (Q1, Q3)	19.09 (16.45, 22.13)	18.85 (17.00, 20.45)	18.16 (15.97, 20.53)	17.85 (16.06, 21.00)	22.35 (20.50, 24.63)	18.49 (16.30, 20.41)	χ^2^ = 8.16#	0.086
FRES, M (Q1, Q3)	17.00 (8.00, 34.00)	17.50 (12.75, 29.50)	13.50 (8.00, 30.75)	26.00 (13.75, 41.75)	10.00 (4.50, 16.50)	27.50 (11.25, 34.25)	χ^2^ = 8.33#	0.08
GFOG, M (Q1, Q3)	16.35 (14.25, 18.30)	16.05 (15.02, 17.32)	16.65 (14.78, 18.30)	14.90 (13.47, 17.62)	17.40 (16.05, 19.40)	16.45 (13.95, 18.50)	χ^2^ = 3.84#	0.427
FKGL, M (Q1, Q3)	16.26 (14.59, 19.21)	16.40 (15.51, 18.14)	15.88 (14.89, 19.09)	15.15 (14.05, 17.88)	19.39 (18.09, 21.09)	15.48 (13.59, 18.49)	χ^2^ = 8.52#	0.074
CL, M (Q1, Q3)	16.63 (14.76, 18.21)	16.27 (15.13, 18.36)	16.71 (15.26, 17.39)	15.52 (12.68, 17.93)	17.70 (16.57, 18.98)	16.50 (14.60, 17.36)	χ^2^ = 7.37#	0.118
SMOG, M (Q1, Q3)	14.62 (12.48, 16.26)	14.59 (12.45, 15.23)	14.34 (13.11, 16.34)	13.12 (12.20, 15.05)	16.26 (15.55, 17.70)	13.91 (12.26, 16.76)	χ^2^ = 9.82#	0.044
LW, M (Q1, Q3)	53.00 (49.00, 59.00)	52.50 (48.00, 57.25)	54.00 (50.50, 59.00)	54.00 (50.50, 57.25)	49.50 (47.00, 53.50)	58.00 (53.00, 63.50)	χ^2^ = 12.82#	0.012

F: ANOVA, #: Kruskal–Wallis test. SD, standard deviation, M, Median, Q1, 1st Quartile, Q3, 3rd Quartile.

### 3.3 Quality analysis

**Figure 1:**
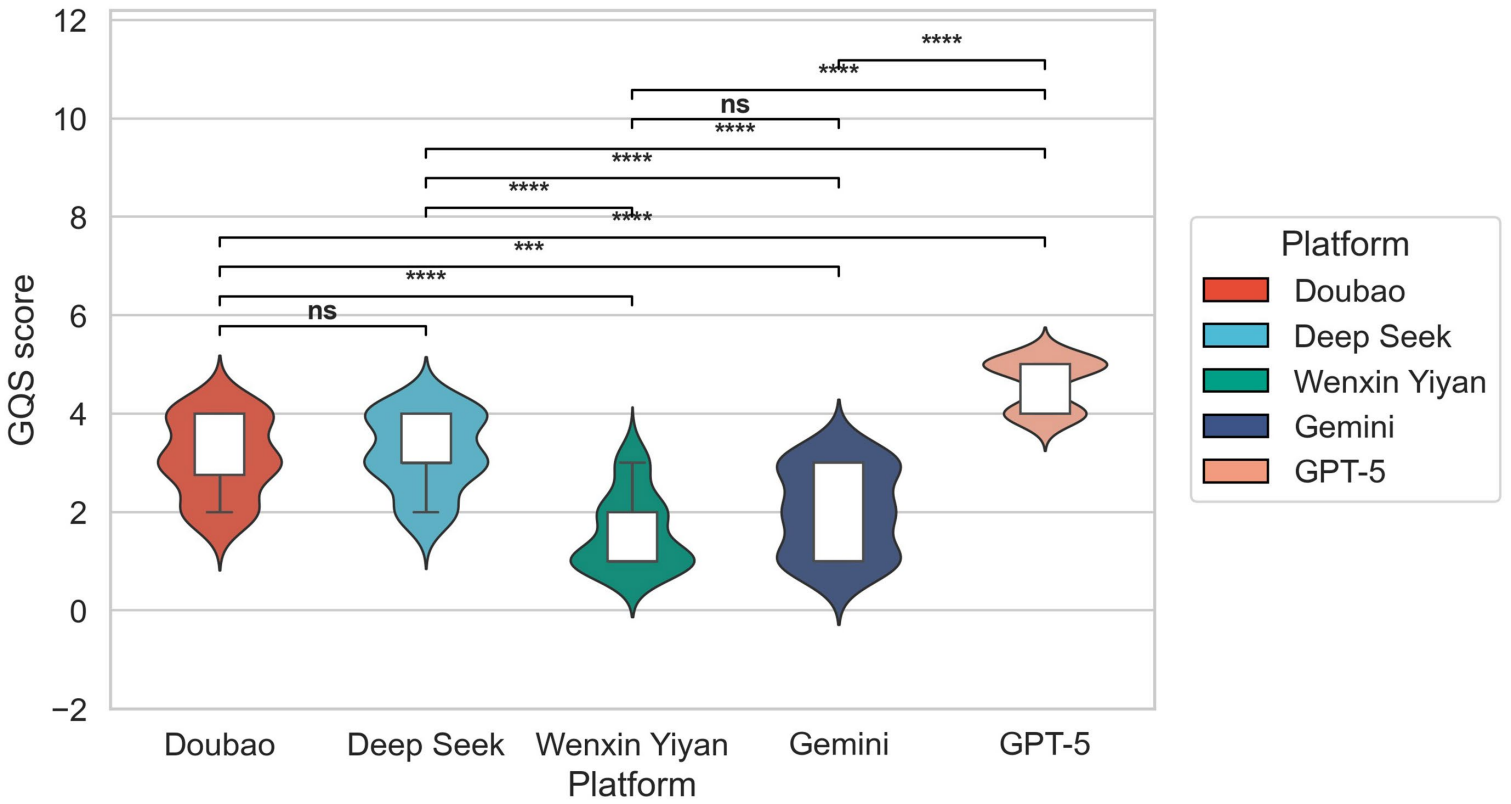
GQS scores of different large models. Significance was assessed using the Kruskal–Wallis test with *post hoc* comparisons (*p < 0.05; **p < 0.01; ***p < 0.001; ****p < 0.0001).

In the GQS score (overall text quality) dimension, platform performance shows significant differentiation: GPT-5 showed the highest GQS score [5.00 (4.00, 5.00)], which was significantly higher than that of all other platforms (*χ*^2^ = 66.52, *p* < 0.001). Wenxin Yiyan showed the lowest GQS score [1.00 (1.00, 2.00)], indicating the weakest core text quality. Doubao [3.00 (2.75, 4.00)] and Deep Seek [3.00 (3.00, 4.00)] fall into the middle score range, with no significant inter-group difference. Gemini [2.00 (1.00, 3.00)] showed higher scores than Wenxin Yiyan but significantly lower scores than GPT-5. These results confirm clear hierarchical variation in core text quality across AI platforms. Across content categories, GQS scores did not differ significantly (*χ*^2^ = 2.54, *p* = 0.637). The distribution of GQS scores across models is illustrated in Figure 1.

**Figure 2:**
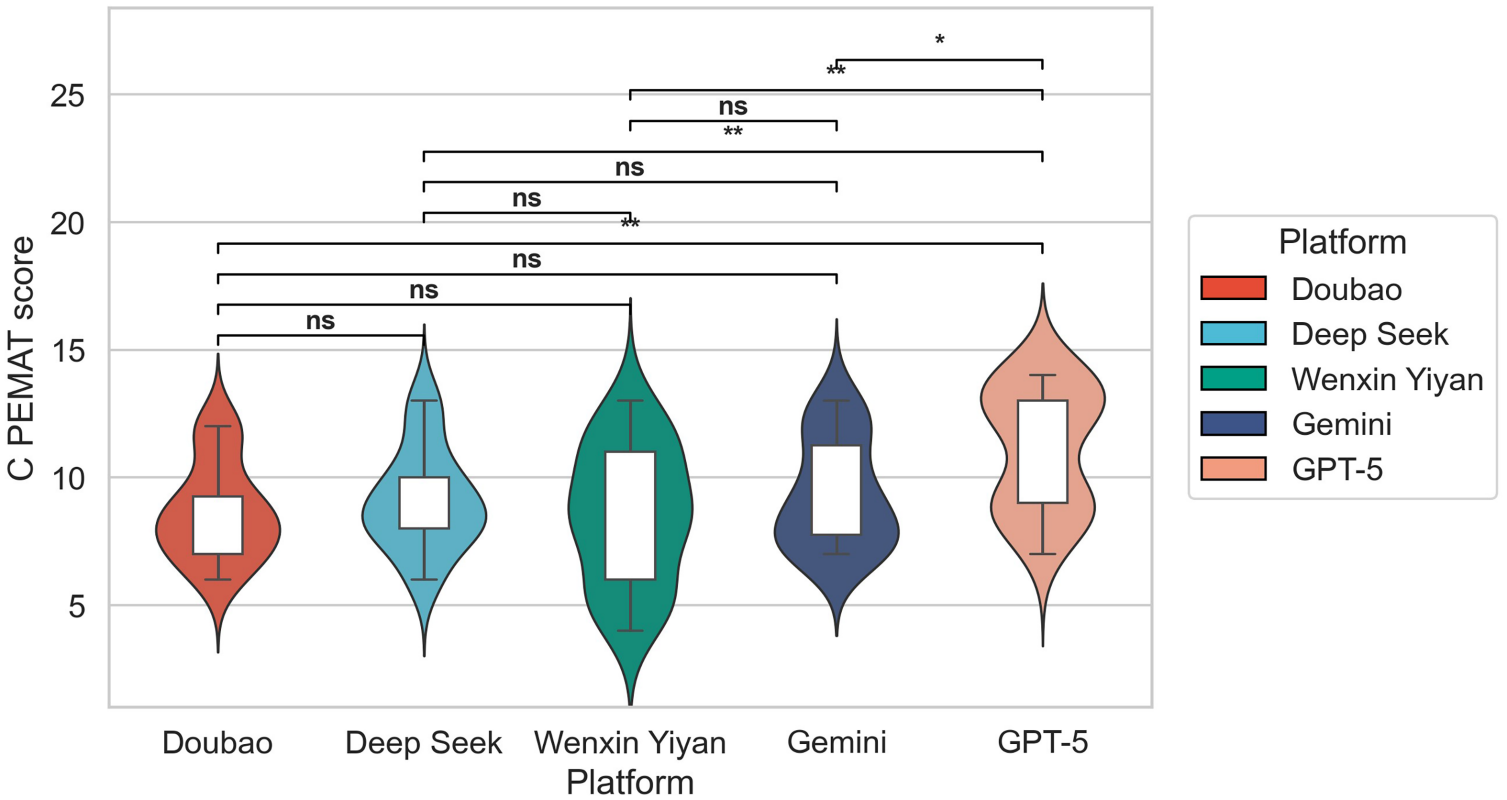
C-PEMAT scores of different large models. Statistical significance was evaluated using one-way ANOVA followed by post hoc testing (*p < 0.05; **p < 0.01; ***p < 0.001; ****p < 0.0001).

Consistently, in the C-PEMAT scoring dimension (assessing patient education suitability), platform performance exhibits distinct stratification: GPT-5’s score is significantly higher than that of Wenxin Yiyan, Gemini, and other platforms (statistically marked **/*). Its generated texts stand out in comprehensibility (clarity of information delivery) and operability (feasibility of practical guidance), closely aligning with patients’ cognitive needs and health literacy levels. In contrast, Wenxin Yiyan’s score is notably lower, with weak patient adaptability and substantial result variability. Doubao, Deep Seek, and Gemini fall into the middle score range, showing no significant inter-group differences (marked ns) and comparable adaptability. Overall, AI platforms display clear hierarchical characteristics in patient education adaptability (cognitively aligned, easy to understand and apply), with GPT-5 significantly outperforming other platforms and Wenxin Yiyan ranking the lowest. Differences in C-PEMAT scores are shown in Figure 2.

### 3.4 Correlation analysis

**Figure 3:**
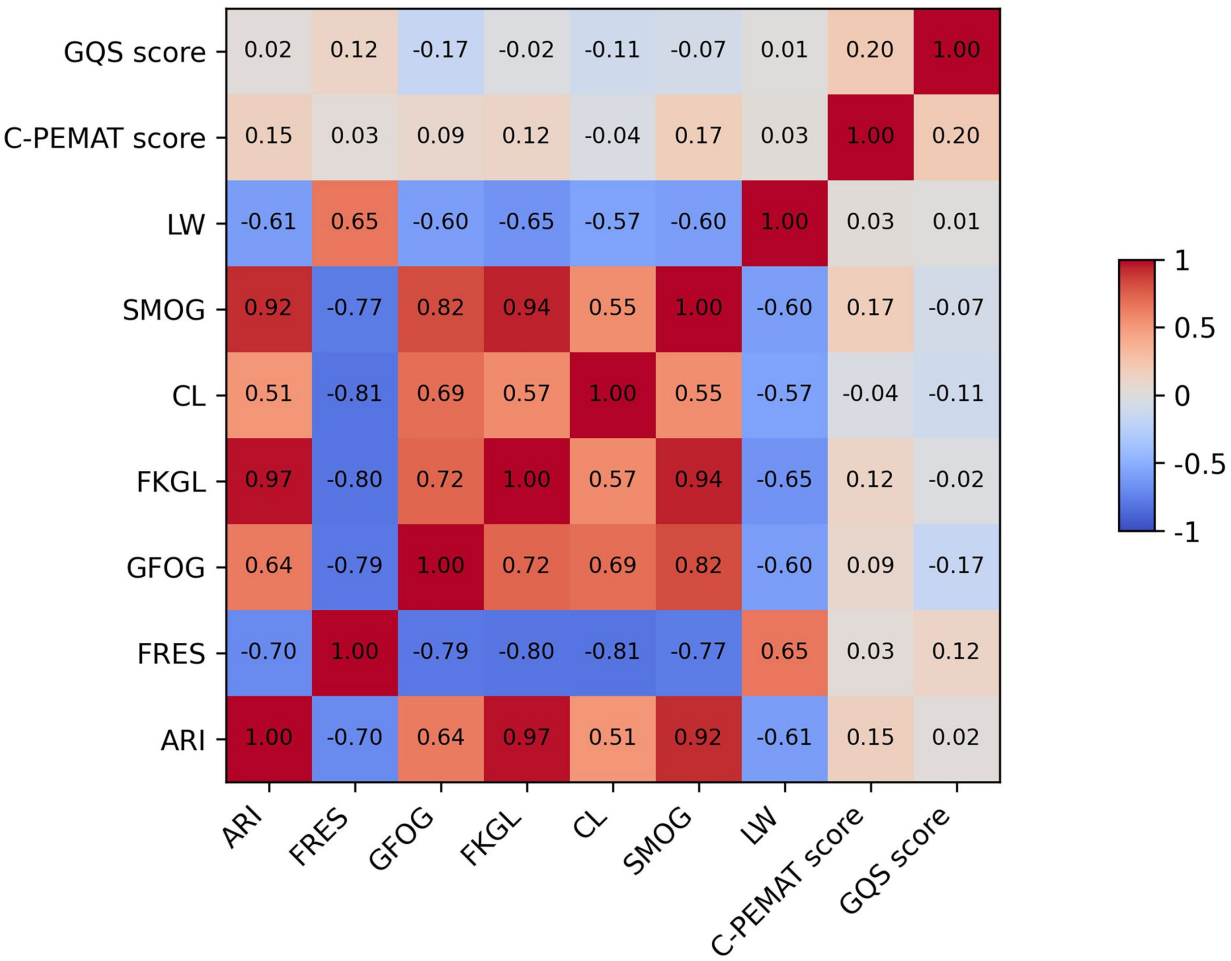
Heatmap of correlations among different indicators. Color intensity represents the strength of correlation: darker colors indicate stronger correlations. Positive values denote positive correlations, and negative values denote negative correlations. Quality indicators include GQS and C-PEMAT; readability indicators include ARI, FRES, GFOG, FKGL, CLI, SMOG, and LW. The heatmap shows strong internal consistency among readability metrics, whereas weak correlations exist between quality and readability indices.

From the correlation distribution in the heatmap (Figure 3), core quality indicators (GQS, C-PEMAT) show weak correlations with readability metrics. Figure 3 presents pairwise correlation coefficients among all quality and readability indicators, where color intensity represents the strength of correlation. Darker red indicates stronger positive correlation, while darker blue indicates stronger negative correlation. This visualization helps readers intuitively distinguish the magnitude and direction of relationships between variables. Correlation relationships among all indicators are visualized in Figure 3. While readability indicators exhibit significantly tight internal associations: extremely strong positive correlations are observed (e.g., ARI & FKGL: 0.97; SMOG & FKGL: 0.94; GFOG & SMOG: 0.82), indicating highly consistent variation trends across text difficulty metrics. In contrast, FRES (readability) shows strong negative correlations with difficulty indicators like ARI (−0.70) and GFOG (−0.79), aligning with the logic that “higher text difficulty corresponds to lower readability.” These results suggest that readability metrics can serve as collaborative tools for evaluating “text reading difficulty,” but their direct impact on “text quality” is limited—"quality” and “readability” are relatively independent dimensions: quality is corely supported by factors such as information accuracy, while readability is dominated by linguistic complexity (vocabulary, sentence structure). Meanwhile, weak correlations exist between quality indicators and some readability metrics: C-PEMAT shows a weak positive correlation with ARI (0.15), implying a marginal effect of text difficulty on patient education suitability; GQS has a weak positive correlation with FRES (0.12), reflecting a slight increase in overall quality with moderately higher readability—these patterns reveal a faint linkage between quality and specific readability indicators.

## 4 Discussion

This study systematically assessed the reliability, quality, readability, and performance variability of HDP patient education content generated by five mainstream large language models (LLMs: Doubao, DeepSeek, Wenxin Yiyan, Gemini, GPT-5). HDP affects roughly 10% of pregnancies globally and ranks among the leading causes of maternal, fetal, and neonatal morbidity and mortality—with the quality of early intervention and health education directly shaping pregnancy outcomes (5, 24). By focusing on two core variables (LLM type and health education content category), we address a critical gap in maternal and child health communication: reconciling AI’s expanding application potential with clinical concerns over output reliability, readability, and alignment with patient health literacy. This work thus provides empirical support for the targeted use of LLMs in obstetric health education.

Readability, a core measure of text comprehensibility, is pivotal to effective health information delivery (25, 26). Complex, lengthy sentences erode reader confidence, whereas short sentences (8–10 words) enhance the efficient spread of health knowledge (12). Today, the internet serves as the primary source of health information for the public: 72% of U. S. adults search for health-related content online, yet the average adult reads at only a 7th–8th grade level (27). Approximately 43 million individuals have low literacy, 8.4 million of whom are illiterate—leaving them unable to interpret basic health information (28). While major health authorities recommend patient education materials tailored to low reading levels, existing online resources and AI-generated content often exceed this standard (21, 22). Most are overly generalized, overlooking individual differences in cultural background and cognitive ability, and thereby creating barriers to health communication.

Performance differences across models follow the general trend of LLM iteration: newer models trained on more precise corpora tend to produce more professional texts. GPT-5 outperformed all other tested models significantly in both core quality (GQS: 5.00 [4.00, 5.00]) and patient education appropriateness (C-PEMAT: 11.10 ± 2.40) (Figure 1). Its optimized architecture facilitates more accurate integration of complex medical information and translation into patient-friendly language—underscoring the potential value of high-performance LLMs for medical text generation (29). This finding reinforces that LLMs are not interchangeable in clinical communication. A recent benchmarking study evaluating five large language models for biomedical communication also reported a consistent three-tier performance hierarchy: GPT-5 achieved the highest scientific quality and patient education suitability, while domestic models including Wenxin Yiyan showed relatively lower reliability and stability (30). Previous research on warfarin counseling also demonstrated that LLMs differ in scientific accuracy and user-friendliness, with ChatGPT providing more accurate responses and Gemini offering clearer, more accessible explanations, which further supports our conclusion of significant performance stratification among models in medical health education (31). Wenxin Yiyan exhibited notably poor quality metrics (GQS: 1.00 [1.00, 2.00]; C-PEMAT: 8.50 ± 2.69), a red flag that widely available models may produce unreliable or patient-misaligned content. Such outputs not only heighten the risk of medical misinformation but also lose practical value when phrased in ways disconnected from patient understanding—echoing recent research highlighting LLMs’ “hallucination” tendencies and inconsistent accuracy in medical contexts (13, 32, 33). In contrast, Doubao’s “high fluency, low complexity” profile (median FRES: 31.50) suits populations with low health literacy—a group at elevated risk of adverse HDP outcomes—aligning closely with health authority guidelines for accessible patient education materials.

Content-specific analysis yielded valuable insights: “Prevention and Prognosis” and “Treatment and Management” achieved the highest C-PEMAT scores (11.20 ± 1.82 and 10.75 ± 2.02, respectively) (Figure 2), suggesting the tested LLMs perform relatively stronger in generating structured, actionable forward guidance—well-matched to the core needs of HDP clinical education. Concern arises, however, from the low score for “Etiology and Risk Factors” (8.00 ± 1.78). HDP’s complex, multi-dimensional etiology demands precise explanations of pathophysiological mechanisms balanced with clinical clarity; describing factors like genetic-environmental interactions also requires reconciling professionalism with accessibility—posing greater challenges to AI’s semantic understanding and information translation. While GQS scores remained stable across themes (χ^2^ = 2.54, *p* = 0.637), indicating consistent perceived professionalism, this does not ensure patient comprehensibility or usability—emphasizing the need to evaluate both quality and readability of LLM outputs. A prior pilot study on aortic stenosis also demonstrated that LLMs vary substantially in improving the readability of cardiovascular patient education materials, further supporting our observation that model selection is critical for accessible obstetric health communication (34).

Correlation analysis revealed a key insight: quality and readability are relatively independent (Figure 3). Readability indices showed strong coherence (e.g., ARI-FKGL correlation coefficient: 0.97) but weak associations with GQS and C-PEMAT (r ≤ 0.15). Highly readable texts may contain factual errors, while overly complex high-quality content (e.g., Gemini’s median GFOG: 17.60) loses clinical utility. Optimal AI-generated health education must therefore excel in both factual accuracy and linguistic accessibility—consistent with the principle that medical text evaluation balances “groundedness” (factual correctness) and readability. This points to clear optimization paths: tailor language complexity rather than prioritizing one dimension alone.

While our study employed standardized, single-turn prompts to ensure consistency across models, clinical practice frequently uses guided or structured prompts. Recent work suggests that well-designed prompts can improve the accuracy, clarity, and clinical relevance of outputs generated by large language models. For health education related to gestational hypertension, personalized and structured prompts may help balance scientific rigor with readability, particularly for patients with limited health literacy. Future studies should move beyond descriptive analyses and directly compare different prompting strategies to refine AI-assisted obstetric health communication.

In summary, LLMs hold significant potential for HDP health education but require targeted model selection and content refinement based on clinical needs. In practice, high-performance LLMs can serve as valuable adjuncts to health education—personalizing content and adapting language for varying health literacy levels to boost patient HDP awareness, treatment adherence, and self-management (35, 36). Critically, they cannot replace clinicians’ professional diagnosis or face-to-face consultation.

Three core challenges merit focus: first, balancing clarity—particularly for weak themes like “Etiology and Risk Factors”—through prompt optimization and clinical expert review; second, matching content to diverse health literacy levels by adjusting language complexity dynamically; third, mitigating ethical and privacy risks. Given that health information involves sensitive patient data, LLM usage protocols must be clear, patients informed of information sources and generation logic, and privacy protected to build trust.

Notably, most existing research focuses on English-language AI health content, leaving LLM performance in multilingual, cross-cultural contexts undervalidated—a gap to address in future work. Upcoming studies should validate models in real-world settings, incorporate feedback from HDP patients and obstetricians, and quantify impacts on health behaviors and pregnancy outcomes. Exploring AI-driven multimodal tools (e.g., videos, interactive graphics, virtual reality) could also enhance the delivery of complex medical knowledge, surpassing traditional text to improve patient understanding and adherence—driving deeper integration of AI into obstetric health education.

## 5 Conclusion

LLMs including Doubao, DeepSeek, and GPT-5 are increasingly effective at delivering medical information on HDP, opening a valuable opportunity to raise HDP awareness and boost patient satisfaction. That said, persistent concerns surround the readability, quality, and reliability of LLM-generated content.

Using LLM type and health education topic as core variables, this study systematically assessed the quality and readability of HDP-related texts, employing metrics such as C-PEMAT, GQS, and a suite of readability indices. Our findings show that LLM type is the primary driver of core text quality: GPT-5 outperformed all other models significantly in both patient education appropriateness and overall quality, consistently producing high-quality content across diverse themes. Content category primarily influenced readability: technically focused topics like “Treatment and Management” yielded texts with greater linguistic complexity and lower readability, yet had no meaningful impact on overall quality. Further analysis revealed that text quality and readability are largely independent domains, with only subtle associations: moderate use of professional terminology strengthens content quality, whereas excessive complexity hinders patient understanding.

These findings highlight the performance differences among LLMs and underscore the importance of matching readability levels to audience health literacy for effective obstetric health communication. Future research should expand the range of models and topics, integrate patient feedback and clinical outcome data, and strengthen the evidence base for LLM applications in health science communication.

## 6 Limitation

While this study provides empirical evidence for artificial intelligence-assisted health education text generation, it has five key limitations. First, the sample size is constrained (total *n* = 100), covering only five mainstream large language models (LLMs) and five health education topic categories. This narrow scope may not fully capture the characteristics of other models (e.g., the GPT series, iFlytek Spark) or specialized topics such as rare diseases and genetic disorders. Second, the assessment relies solely on objective indicators, without integrating patients’ subjective experience data (e.g., text comprehension scores, operational implementation rates) or clinical evaluations by medical professionals. This makes it challenging to comprehensively reflect the actual application effectiveness of the generated texts. Third, the study did not account for the impact of patients’ individual differences (e.g., age, educational background, health literacy level) on text acceptance, which means the pertinence of the proposed optimization strategies requires further enhancement. Fourth, model outputs were collected at a single time point (November 25, 2025). LLMs are frequently updated, which may cause temporal shifts in response quality and readability; future studies should include longitudinal assessments. Fifth, this study remains largely descriptive in design and did not experimentally manipulate prompt strategies. Although standardized prompts ensured fair cross-model comparison, real-world clinical applications often use guided, constrained, or role-based prompts. Future research should conduct controlled experiments to compare how different prompting approaches affect the quality and readability of AI-generated health education materials. Sixth, due to the lack of validated and widely accepted readability indices for Chinese medical texts, all questions and responses required dual translation (Chinese-to-English and English-to-Chinese). Although the entire translation process was completed by a professional obstetrics and gynecology specialist to ensure consistency, potential linguistic differences caused by translation may still exist and should be considered when interpreting the results. Nevertheless, all outputs were processed in an identical manner, thus preserving full cross-model comparability. Seventh, all models were evaluated using English prompts and outputs. Doubao, Deep Seek, and Wenxin Yiyan are Chinese-preferred large language models primarily optimized for Chinese understanding and generation. Their performance on English medical questions may be inherently disadvantaged compared with internationally developed models such as GPT-5 and Gemini, which could introduce systematic differences unrelated to actual clinical quality. This language-origin disadvantage should be considered when interpreting between-group comparisons.

## 7 Strength of the study

This study establishes a rigorous multi-dimensional evaluation framework, integrating C-PEMAT, GQS, and readability indices to systematically assess LLM-generated texts on HDP. It simultaneously examines the impacts of LLM types and content categories, offering targeted implications for maternal health communication. Correlation analysis clarifies the independence of quality and readability, laying robust empirical groundwork for LLM applications in health science communication.

## Data Availability

The original contributions presented in the study are included in the article/supplementary material, further inquiries can be directed to the corresponding author.

## Footnotes

1
https://www.doubao.com/chat/

2
https://chat.deepseek.com/

3
https://yiyan.baidu.com/

4
https://gemini.google.com/

5
https://chatgpt.com/

6
http://readabilityformulas.com/
